# Impact of Organic and Inorganic Fertilizers Application on the Phytochemical and Antioxidant Activity of Kacip Fatimah (*Labisia pumila* Benth)

**DOI:** 10.3390/molecules180910973

**Published:** 2013-09-05

**Authors:** Mohd Hafiz Ibrahim, Hawa Z. E. Jaafar, Ehsan Karimi, Ali Ghasemzadeh

**Affiliations:** 1 Department of Biology, Faculty of Science, University Putra Malaysia, Serdang 43400, Selangor, Malaysia; 2 Department of Crop Science, Faculty of Agriculture, University Putra Malaysia, Serdang 43400, Selangor, Malaysia; E-Mails: hawazej@gmail.com (H.Z.E.J.); ehsan_b_karimi@yahoo.com (E.K.); upmali@yahoo.com (A.G.)

**Keywords:** medicinal plants, *Labisia pumila*, fertilizer effects, plant secondary metabolites, antioxidant activity

## Abstract

A study was conducted to compare secondary metabolites and antioxidant activity of *Labisia pumila* Benth (Kacip Fatimah) in response to two sources of fertilizer [*i.e.*, organic (chicken dung; 10% N:10% P_2_O_5_:10% K_2_O) and inorganic fertilizer (NPK green; 15% N, 15% P_2_O_5_, 15% K_2_O)] under different N rates of 0, 90, 180 and 270 kg N/ha. The experiment was arranged in a randomized complete block design replicated three times. At the end of 15 weeks, it was observed that the application of organic fertilizer enhanced the production of total phenolics, flavonoids, ascorbic acid, saponin and gluthathione content in *L. pumila*, compared to the use of inorganic fertilizer. The nitrate content was also reduced under organic fertilization. The application of nitrogen at 90 kg N/ha improved the production of secondary metabolites in *Labisia pumila*. Higher rates in excess of 90 kg N/ha reduced the level of secondary metabolites and antioxidant activity of this herb. The DPPH and FRAP activity was also highest at 90 kg N/ha. The results indicated that the use of chicken dung can enhance the production of secondary metabolites and improve antioxidant activity of this herb.

## 1. Introduction

Medicinal plants are used to cure many ailments that are either non-curable or seldom cured through modern systems of medicine. Approximately 80% of the World population depends on medicinal plants for their health and healing [1]. Societal motivations to use herbs are increasing due to concern about the side effects of synthetic drugs. Many botanicals and some dietary supplements are good sources of antioxidants and anti-inflammatory compounds [2]. *Labisia pumila* Benth*.*, commonly called ‘Kacip Fatimah’, of the family Myrsinaceae is an important medicinal herb which has been used in traditional Malay medicine since time immemorial. The roots, leaves or the whole plant are boiled in water [3,4] and the water taken orally to facilitate labor, shrink the uterus and improve menstrual irregularity and for post-partum medicine [5,6]. Its usage for women’s health may be related to its phytoestrogen effects and content of chemicals with similar structure to estrogen [7]. *Labisia pumila* extract contains flavanoids, phenolics and various bioactive volatile compounds [8]. Chemical analysis of its roots revealed three novel metabolites, demethylbelamcandaquinone B, fatimahol and dexylo-primulanin, together with 21 known compounds, including epoxyoleanane glycosides, alkenated phenolics, cerebroside, glycerogalactolipids, and lipids [9].

For healthy growth and optimal yield, nutrients must be available to plants in correct quantity, proportion and in a usable form at the right time. To fulfill these requirements, chemical fertilizers and/or organic manures are needed. Fertilization has been reported to have an influence on the phyto-nutritional quality of crops. Inorganic fertilizer is said to reduce the antioxidant levels, while organic fertilizer has been proven to enhance antioxidant content in plants [10]. Applying fertilizers, particularly in the inorganic form, in excess of plant requirements can increase the chances of fertilizer loss and environmental pollution. Organic manures, apart from improving physical and biological properties of soil, help in improving the efficiency of chemical fertilizers [11]. Organic manures such as farmyard and poultry manure are known to improve the physical, chemical and biological conditions of soil and ensure sustainable soil health [12].

In the past, agricultural production was focused on maximizing the quantity of crop produced for commercial markets. Hence, compound fertilizer has been used as a common agricultural practice. However, recently health conscious consumers are interested in optimizing the nutritional composition with minimal chemical residues on foods produced through environmentally friendly agricultural practices [13]. Substituting chemicals with organic fertilizers is one of the common principles in this production system. Inorganic fertilizers have had significant effects on World crop production and are essential components of today’s agriculture. Estimates show that agricultural production is raised by 50% as a result of chemical fertilizers and 60% of the population owes its nutritional survival to nitrogen (N) fertilizers [14]. However, of the total applied N, less than 50% is recovered in the soil–plant system, while the remainder is lost to the environment [15]. 

Plants can take up nitrogen (N) either as inorganic ions (NH_4_^+^ or NO_3_^−^), or as organic N [16,17,18]. Gao *et al.* [18] observed that application of high NH_4_^+^ and low NO_3_^−^ levels resulted in improved fruit quality. There is a scarcity of data on the effect of the form of N on the production of secondary metabolites in plants [19]. According to the C/N balance hypothesis, when N is readily available, plants will primarily make compounds with high N content (e.g., proteins for growth). When N availability is limited, metabolism changes more towards carbon-containing compounds such as starch, cellulose, and non-N-containing secondary metabolites such as phenolics and terpenoids [20]. The relative differences in the release of nutrients from various fertilizers could lead to different C/N ratios in plants and this in turn could lead to a difference in the production of secondary metabolites [17]. Recently, Hakkinen and Torronen [21] compared the phenolic content in three cultivars of strawberries grown organically and conventionally. They reported that only one cultivar grown under organic conditions showed higher levels of phenolics than its inorganically grown counterparts. Asami *et al.* [22] reported significantly higher total phenolics in marionberries grown with organic fertilizer as compared with inorganic fertilizer. Weibel *et al.* [23] indicated that the phenol (mainly flavonols) content of organically grown apple cultivars was 19% higher than in apples grown using inorganic fertilizers. These results suggest that the usage of organic fertilizers can enhance the production of plant secondary metabolites.

The fertilizer impact on vegetative growth is well documented. However, the effect of fertilizer rates and sources on phytochemical quality in *L. pumila* Benth is lacking. There have been numerous studies that have investigated the effects of various environment factors on plant primary and secondary metabolites of this herb [24,25,26,27,28,29,30,31,32,33,34], but only limited studies covering the response of secondary metabolites and antioxidant activities under different fertilizer sources and rates. Hence, the objective of this study was to examine the effect of fertilizer source (chicken dung and NPK green) and fertilizer rates (0, 90, 180 and 270 kg N/ha) on the production of secondary metabolites (total phenolics, flavonoid, phenolics, ascorbic acid, saponin) and antioxidant activity (DPPH and FRAP) of *L .pumila* var *alata* seedlings. The relationships between these parameters were also investigated.

## 2. Results and Discussion

### 2.1. Total Phenolics and Flavonoids

Total phenolics and flavonoids were influenced by fertilizer source and fertilization rates (*p* ≤ 0.05). It was observed that that application of organic fertilizer increased the production of total phenolics and flavonoids contents in *L. pumila* Benth. (Table 1). Total phenolics and flavonoids were enhanced 12% and 22%, respectively, in the organic fertilizer treatment compared to inorganic fertilization. It was apparent that optimum fertilization ocurred at 90 kg N/ha, where it was observed that total phenolics and flavonoids had the highest values (total phenolics = 1.32 mg/g gallic acid dry weight, and total flavonoids = 0.81 mg/g rutin dry weight). Increased fertilization rates above 90 kg N/ha to 180 and 270 kg N/ha resulted in a decrease in the total phenolics and flavonoid contents. Ferry *et al.* [35] and Ranelletti *et al*. [36] have also shown that these phenolics and flavonoids had anticancer activities, and that they were able to inhibit cancer cell growth. Gallic acid and rutin were reported to have high scavenging activity and act as treatments for diabetes, albuminaria, psoriasis and external haemorrhoids [37,38]. Some studies have reported that gallic acid and rutin play an important role in the prevention of cancer [39]. The present results suggest that the application of organic fertilizers can enhance *L. pumila* antimicrobial and anticancer activities. The results indicated that total phenolics and flavonoid had a positive significant correlation with soluble sugar (total phenolics = r^2^ = 0.912; total flavonoid r^2^ = 0.923; *p* ≤ 0.05) (Table 2). This indicated that under low fertilization rates there will be an accumulation of soluble sugar that might enhance the total phenolics and flavonoids in *Labisia pumila*. Previous studies done on *Labisia pumila* have indicated that carbohydrate and production of total phenolics and flavonoid was positively correlated and this was also observed in the present study [24,25,26,27,28,29,30,31,32,33,34].

**Table 1:** Total phenolics, flavonoids, vitamin c and saponin concentrations in leaf tissue of *L. pumila* in response to fertilizer rates and source.

Treatments	Total phenolics (mg gallic acid/g dry weight	Total flavonoids (mg rutin/g dry weight)	Vitamin C (mg/g fresh weight)	Saponin (mg diosgenin/g dry weight)
*Fertilizer sources*				
Organic (Chicken dung)	1.10a	0.76a	0.061b	38.16a
Inorganic (NPK green)	0.98b	0.62b	0.078a	32.17b
*Fertilizer rates (kg N/ha)*				
0	1.22b	0.81b	0.072b	47.21b
90	1.32a	0.98a	0.089a	58.14a
180	1.02c	0.72c	0.060c	32.18c
270	0.87d	0.41d	0.047d	20.17d
Source × Rate	ns	ns	ns	ns

ns: non significant at *p* ≤ 0.05. Means followed by the same letters are not significantly different by DNMRT test (*p* ≤ 0.05).

**Table 2:** The correlationship among the parameters recorded in the study (total phenolics, total flavonoids, vitamin c, saponin, soluble sugar, nitrate, gluthathione, DPPH and FRAP).

Parameters	1	2	3	4	5	6	7	8	9
1. T. phenolics	1.000								
2. T. flavonoids	0.899 *	1.000							
3. Vitamin C	0.768 *	0.777 *	1.000						
4. Saponin	0.912 *	0.813 *	0.776 *	1.000					
5. Soluble sugar	0.912 *	0.923 *	0.954 *	0.914 *	1.000				
6. Nitrate	0.212	0.123	0.231	0.321	0.216	1.000			
7.Gluthathione	0.914 **	0.924 *	0.889 *	0.824 *	0.778 *	0.123	1.000		
8. DPPH	0.912 *	0.915 *	0.911 *	0.976 *	0.918 *	0.126	0.908 *	1.000	
9. FRAP	0.899 *	0.917 *	0.931 *	0.876 *	0.854 *	0.421	0.912 *	0.987 *	1.000

*,** significant at *P* ≤ 0.05 or *P* ≤ 0.01.

### 2.2. Ascorbic Acid and Saponin Content

The same trend was also observed for ascorbic acid and saponin contents in *Labisia pumila*. It was observed that ascorbic acid and saponin content was maximized at 90 kg N/ha. The increase in fertilization rates (0 > 270 kg N/ha) reduced the production of ascorbic acid and saponin contents in this plant. The reduction in ascorbic acid and saponin content under high fertilization was also reported in other studies [40,41,42]. Saponin content was highest under organic fertilization that recorded 38.16 mg/g fresh weight, and lowest with inorganic fertilization which only registered 32.17 mg/g fresh weight. However, ascorbic acid was found to be highest with inorganic fertilization. The usage of inorganic fertilization increased the ascorbic acid content by 27% compared to the usage of organic fertilizer. It was observed that organic fertilization enhanced the production of total phenolics, flavonoids and saponin contents in *L. pumila* (Table 2). The increase in production of secondary metabolites under organic fertilization in the present study might be due to high in micronutrients content in the organic fertilizer used. Plants grown under organic agricultural conditions are reported to have higher micronutrient content in more cases than conventionally grown plants [23,24]. Considering the fact that some of chemical reactions in cells involve minor elements, either directly or indirectly, this could explain why organic-fertilized plants exhibited higher production of secondary metabolites [43].

### 2.3. Soluble Sugar and Nitrate

Soluble sugar content was influenced by fertilizer type and rates (*p* ≤ 0.05). It was observed that soluble sugar content was higher under organic fertilization compared to inorganic fertilization. At the end of 15 weeks the soluble sugar content in the organic fertilizer treatment was 97.2 mg/g dry weight compared to 92.10 mg/g dry weight recorded with the inorganic fertilization. Less soluble sugar was produced in 0 kg N/ha (98.10 mg/g dry weight), 180 kg N/ha (71.25 mg/g dry weight) and 270 kg N/ha (62.31 mg/g dry weight) treatments compared to fertilization at 90 kg N/ha, which produced the highest soluble sugar accumulation (102.70 mg/g dry weight). This phenomenon was also observed by other researchers [44,45,46]. The accumulation of soluble sugar under low nitrogen fertilization might be due to reduction in sink size of the plant when nitrogen is limited, hence reducing the translocation of carbohydrate to the other plant parts [47]. The current results showed that high application rates of fertilizer increased the nitrate content in leaf sap samples (Table 3). It was also observed that the nitrate content was statistically lower in the treatment with organic fertilizer compared to inorganic fertilizer. The nitrate content with inorganic fertilizer was 13% more than with organic fertilizer. Nitrate has been attributed to negative effects to human health. Toxicity of nitrate to human can be manifested by headaches, syncope, vertigo and discoloration that manifest in fingers or lips [48]. The results indicate that the usage of organic fertilizer can minimize the nitrate content in plants. Organic fertilizer contains nitrogen bound to organic material that is slowly released [49]. Inorganic fertilizer which is absorbed rapidly into the plant usually have higher nitrosamines which have been associated with chronic diseases such as leukemia and gastrointestinal cancer [50]. The nitrate levels that acceptable to human are 10–100 ppm in drinking water. Intake more than 100 ppm would induce the toxic effects [51]. In the current result, the nitrate value for organic and non organic was higher than acceptable recommended levels (>100 ppm). However, most vegetables and fruits contained nitrate levels from 20–200 ppm [52]. Usually, the processing of food (e.g., chopping, grinding and heating) would reduce the nitrate content and thus reduce the negative effects [53]. The current result implies that the application of organic fertilizer to *L. pumila* can reduce the nitrate content and plays an important role in the production of healthier herbal products [54].

**Table 3:** Total soluble sugar, nitrate and gluthathione concentration in leaf tissue of *L. pumila* in response to fertilizer rates and source.

Treatments	Soluble sugar (mg sucrose/g dry weight)	Nitrate (ppm)	Gluthathione (nmol/g dry weight)
*Fertilizer sources*			
Organic (Chicken dung)	97.21a	157.21b	616.71b
Inorganic (NPK green)	90.02b	178.79a	632.16a
*Fertilizer rates (kg N/ha)*			
0	98.17b	112.31b	777.21b
90	102.72a	137.21a	831.28a
180	71.25c	198.21c	621.31c
270	62.31d	211.31d	522.18d
Source x Rate	ns	ns	ns

ns: non significant at *p* ≤ 0.05. Means followed by the same letters are not significantly different by DNMRT test (*p* ≤ 0.05).

### 2.4. Gluthathione Content

The gluthathione levels were influenced by fertilizer source and rates (*p* ≤ 0.05). The production of gluthathione fertilized at 270 kg N/ha was significantly lower than other fertilization rates. At the end of 12 weeks of measurement, gluthathione for 0, 90 and 180 kg N/ha was 771.21, 831.28 and 621.31 nmol compared to only 522.18 nmol for 270 kg N/ha. The gluthathione levels at 0, 90 and 180 kg n/ha were 47, 59 and 19% higher than with fertilization at 270 kg N/ha. GSH is a tri-peptide composed of cysteine, glutamic acid and glycine and is the most abundant non-protein thiol in the cells. Its active group is the thiol (-SH) of cysteine. GSH is maintained in the reduced state. The GSH plays an imperative role in the stabilization of many antioxidant enzymes [55]. Additionally, as an antioxidant scavenger it serves as a substrate for dehydroascorbate (DHAsA) reductase and is also directly reactive with free radicals including the hydroxyl radical to prevent the inactivation of enzymes by oxidation of an essential thiol group [56]. In the present study, we found that reduced N fertilization increased GSH. High GSH is necessary for several physiological functions, including activation and inactivation of redox-dependent enzyme systems and regeneration of the cellular antioxidant ascorbic acid under oxidative conditions [57,58]. Usually, the increase in GSH with reduced N fertilization is associated with an increase in antioxidant properties [59]. In the current study, it was shown that GSH had a strong positive relationship with total phenolics, flavonoids, ascorbic acid and saponin content (Table 3). The results showed that the increase in antioxidative properties of *L. pumila* under low nitrogen fertilization might be due to an increase in production of total phenolics, flavonoids, ascorbic acid, saponin, and that GSH activity can increase the antioxidant capacity of this plant under this condition [60,61].

### 2.5. DPPH and FRAP

The effects on DPPH are attributed to fertilizer source and rates of nitrogen levels (*p* ≤ 0.05; Table 4). At 380 µg/mL, the DPPH antioxidant activity recorded the highest value (63.18%) at 90 kg N/ha followed by the 0 kg N/ha (57.12%), 180 kg N/ha (47.22%), and the least in the 270 kg N/ha treatment (38.99%). The DPPH values were found to be highest under organic fertilization (47.28%) and lowest under inorganic fertilization (40.21%). The results imply that the usage of organic fertilizer can enhance the radical scavenging activity in *L. pumila* and high fertilizer rates could significantly reduce the DPPH radical scavenging activity of the medicinal plant. It must be noted that the DPPH assay principally measures the activity of water-soluble antioxidants [62]. The principle of this method is that in the presence of a molecule consisting of a stable free radical (DPPH), an antioxidant with the ability to donate a hydrogen atom will quench the stable free radical, a process which is associated with a change in the absorption and can be measured spectrophotometrically. Results of the current work also suggest that high N supply was disadvantageous for the improvement of the antioxidant activity of water-soluble antioxidants in *L. pumila*. Besides phenolics and flavonoid compounds, other water-soluble antioxidants in the extracts such as vitamin C could also exert an additive effect on DPPH radical scavenging activity. Studies have shown that a combination of phenolics and ascorbic acid produced a synergistic effect on DPPH radical scavenging activity [63].

**Table 4:** DPPH and FRAP scavenging assay in leaf tissue of *L. pumila* in response to fertilizer rates and source.

Treatments	DPPH scavenging assay (%)	FRAP scavenging assay (µm fe(II)/g dry weight)
*Fertilizer sources*		
Organic (Chicken dung)	47.28a	600.24a
Inorganic (NPK green)	40.21b	528.17b
*Fertilizer rates (kg N/ha)*		
0	57.12b	701.24b
90	63.18a	814.21a
180	47.22c	621.71c
270	41.10d	511.78d
Source × Rate	ns	ns

ns: non significant at *p* ≤ 0.05. Means followed by the same letters are not significantly different by the DNMRT test (*p* ≤ 0.05).

The Ferric Reducing Antioxidant Potential (FRAP) assay measures the total antioxidant power of biological fluids [64]. Total antioxidant power was assessed by the reduction of Fe^3+^ to Fe^2+^, which occurred rapidly with all reductants with half of the reaction reduction potentials above that of Fe^3+^/Fe^2+^. Therefore, the values express the corresponding concentration of electron-donating antioxidants. The FRAP was influenced by fertilizer source and rates (*p* ≤ 0.01), and followed the same trend as with DPPH, where the reducing ability was highest under organic fertilization with the activity maximized at 90 kg N/ha, and was the lowest activity with 270 kg N/ha. The present results indicate that fertilization with low N enabled high abilities to reduce ferric Ions. The antioxidant potential of *L. pumila* was estimated by the ability to reduce 2,4,6-tripyridyl-s-triazine (TPTZ)-Fe(III) complex to TPTZ-Fe(II). The ferric reducing ability (FRAP assay) has been widely used in the evaluation of the antioxidant component of dietary polyphenols [65]. The antioxidant activity is found to be linearly proportionate to the phenolics and flavonoids content [66,67,68,69]. Yen *et al.* [70] reported that the ferric reducing power of bioactive compounds was associated with antioxidant activity. Glenn *et al.* [71] reported a strong positive relationship between total phenolics, flavonoids compounds and antioxidant activity. A similar trend was observed with the current study where total phenolics and flavonoids displayed significantly positive relationships with FRAP activity (r^2^ = 0.899 and r^2^ = 0.917; *p* ≤ 0.05; Table 2). Furthermore, DPPH and FRAP had a significant positive relationship with GSH and ascorbic acid, and thus justifies that high DPPH and FRAP activity in *L. pumila* extracts under organic fertilization at low rates might be due to high accumulation of total phenolics, total flavonoids, gluthatione and ascorbic acid in the plant.

The positive impact of chicken dung (organic fertilizer) applied at the lower rates may be due to increased metabolic activity under these conditions [72]. The usage of organic fertilizer improves soil properties by increasing soil physical, chemical and biological properties, but the usage also prevents soil erosion [73]. The increase in secondary metabolites and antioxidant activity with organic fertilizer might be due to the availability of various other major and minor elements, whereas the inorganic fertilizer used in the current study only supplied the three major elements [Nitrogen (N;15%), Potassium (P_2_O_5_; 15%) and Phosphorous (K_2_O; 15%)]. The current study indicated that supplying *L. pumila* with chicken dung (organic fertilizer) improves the secondary metabolites production and antioxidant activity of this plant especially under low N (<90 kg N/ha).

## 3. Experimental

### 3.1. Experimental Location, Plant Materials and Treatments

The experiment was carried out in a glasshouse complex at Field 2, Faculty of Agriculture Glasshouse Complex, Universiti Putra Malaysia (longitude 101° 44′ N and latitude 2° 58′ S, 68 m above sea level) with a mean atmospheric pressure of 1.013 kPa. The seedlings were planted in a soilless medium containing coco-peat, burnt paddy husk and well composted chicken manure in a 5:5:1 (v/v) ratio in 25 cm diameter polyethylene bags. The media had a pH value of 6.00. Day and night temperatures were maintained at 27–30 °C and 18–21 °C, respectively, with a relative humidity of between 50 to 60%. All the seedlings were irrigated using overhead mist irrigation, given four times a day or when necessary. Each irrigation session lasted for 7 min. Each treatment consisted of seven seedlings, and there were a total of 252 seedlings used in the experiment. Three-month old *L. pumila* var *alata* seedlings were left for a month in the nursery to acclimatize until they were ready for the treatments. When the seedlings had reached 4 months of age they were fertilized with one of two fertilizer sources [*i.e.*, chicken dung based BIO-ORGANIC^®^ organic fertilizer (10% N: 10% P_2_O_5_: 10% K_2_O) and inorganic fertilizer NPK green (15% N, 15% P_2_O_5_, 15% K_2_O)] and were evaluated at different N rates of 0, 90, 180 and 270 kg N/ha. This factorial experiment was arranged in a randomized complete block (RCBD) design with three replications. All the plants were harvested after 15 weeks of treatment.

### 3.2. Determination of Total Phenolics and Flavonoids

The methods used for extraction and quantification of total phenolics and flavonoids contents followed that described in Ibrahim *et al.* [26]. A fixed amount of ground tissue samples (0.1 g) was extracted with 80% ethanol (10 mL) on an orbital shaker for 120 min at 50 °C. The mixture was subsequently filtered (Whatman™ No.1), and the filtrate was used for the quantification of total phenolics and total flavonoids. Folin–Ciocalteu reagent (diluted 10-fold) was used to determine total phenolics content of the leaf samples. Two hundred µL of the sample extract was mixed with Folin–Ciocalteau reagent (1.5 mL) and allowed to stand at 22 °C for 5 min before adding NaNO_3_ solution (1.5 mL, 60 g L^−1^). After two hours at 22 °C, absorbance was measured at 725 nm. The results were expressed as mg g^−1^ gallic acid equivalent (mg GAE g^−1^ dry sample). For total flavonoids determination, samples (1 mL) were mixed with NaNO_3_ (0.3 mL) in a test tube covered with aluminium foil, and left for 5 min. Then 10% AlCl_3_ (0.3 mL) was added followed by addition of 1 M NaOH (2 mL). The absorbance was measured at 510 nm using a spectrophotometer with rutin as a standard (results expressed as mg/g rutin dry sample).

### 3.3. Total Saponin Determination

Total saponin content was determined according to Makkar and Becker [74] based on the vanillin-sulfuric acid colorimetric reaction. The results were expressed as mg diosgenin equivalent per gram dry matter of plant material.

### 3.4. Ascorbic Acid Determination

The ascorbic acid content was measured using a modified method of Davis and Masten [75]. The fresh leaf samples (1 g) were extracted in 1% of phosphate-citrate buffer, pH 3.5 using a chilled pestle and mortar. The homogenate was filtered. The filtrate was added to 1.7 mM 2,6-dichloroindophenol (2,6-DCPIP, 1 mL) in a 3 mL cuvette. The absorbance at 520 nm was read within 10 min of mixing the reagents. The extraction buffer was used as a blank. L-Ascorbic acid was used as a standard. Ascorbic acid was recorded as mg/g L-ascorbic acid in fresh leaves.

### 3.5. Soluble Sugar Determination

Soluble sugar was measured spectrophotometrically using the method of Ibrahim *et al.* [25]. Samples (0.5 g) were placed in 15 mL conical tubes, and distilled water added to make up the volume to 10 mL. The mixture was then vortexed and incubated for 10 min. Anthrone reagent was prepared using anthrone (Sigma Aldrich, St. Louis, MO, USA, 0.1 g) that was dissolved in 95% sulphuric acid (Fisher Scientific, Los Angeles, CA, USA 50 mL). Sucrose was used as a standard stock solution to prepare a standard curve for the quantification of sucrose in the sample. The mixture of ground dry sample and distilled water was centrifuged at a speed of 3400 rpm for 10 min and then filtered to get the supernatant. A sample (4 mL) was mixed with anthrone reagent (8 mL) and then placed in a water-bath at 100 °C for 5 min before the sample was measured at an absorbance of 620 nm using a spectrophotometer (Model UV160U; Shimadzu Scientific, Kyoto, Japan). The total soluble sugar in the sample was expressed as mg/g sucrose in dry sample.

### 3.6. Nitrate Determination

Fresh leaf samples were collected and kept in a refrigerator prior to analysis. The fresh leaves were cut to small pieces and squeezed in a stainless steel press to obtain the sap. Sap was then used to measure nitrate concentration using a Horiba^®^ Cardy Twin Nitrate Meter (Model B-343; Horiba Scientific Inc., Trenton, NJ, USA).

### 3.7. Gluthathione Determination

Total glutathione were determined by reacting plant extracts (0.5 mL) with 50 mM KH_2_PO_4_/2.5 mM EDTA [28]. buffer (pH 7.5), 0.6 mM DTNB [5,5-dithiobis-2-nitrobenzoic acid] in 100 mM Tris-HCl, pH 8.0, 1 unit of glutathione reductase (GR, from spinach, EC 1.6.4.2) and 0.5 mM NADPH. GSH was quantified from the reaction mixture by mixing plant extracts (0.5 mL) with 60 mM KH_2_PO_4_/2.5 mM EDTA buffer (pH 7.5), 0.6 mM DTNB [5,5-dithiobis-2-nitrobenzoic acid] in 200 mM Tris-HCl, pH 8.0. The mixture was incubated at 30 °C for 15 min, and the rate of change in absorbance was determined at 412 nm using a light spectrophotometer (UV-3101P, Labomed Inc., Lincoln, NE, USA).

### 3.8. DPPH Radical Scavenging Assay

The 1,1-diphenyl-2-picryl-hydrazyl (DDPH) used was purchased from Sigma-Aldrich. The DPPH free radical test was conducted using the method of Ghasemzadeh *et al.* [76]. The initial absorbance of DPPH in methanol was measured at 515 nm until the absorbance remained constant. Extracts (40 µL) were added to alcohol solutions of DPPH (3 mL, 0.1 mM). The samples were first kept in a dark place at room temperature and after 30 min the absorbance was measured using a spectrophotometer (U-2001, Hitachi Instruments Inc., Tokyo, Japan) at 515 nm. The percent of inhibition was determined using the formula: Percent of inhibition (%) = [(A_515_ of control − A_515_ of sample)/A_515_ of control] × 100.

### 3.9. Reducing Ability (FRAP Assay)

The ability to reduce ferric ions was measured using a modified method of Karimi *et al.* [77]. An aliquot (200 µL) of the extract with appropriate dilution was added to 3 mL of FRAP reagent (10 parts of 300 mM sodium acetate buffer at pH 3.6, 1 part of 10 mM TPTZ solution and 1 part of 20 mM FeCl_3_·6H_2_O solution) and the reaction mixture was incubated in a water bath at 37 °C. The increase in absorbance at 593 nm was measured after 30 min. The antioxidant capacity based on the ability to reduce ferric ions of the extract was expressed as µM Fe(II)/g dry mass.

### 3.10. Statistical and Correlation Analysis

Data were analyzed using the analysis of variance procedure in SAS version 17. Means separation between treatments was performed using Duncan multiple range test and the standard error of differences between means was calculated with the assumption that data were normally distributed and equally replicated [78,79,80,81,82]. Correlation analysis were analyzed using Pearson correlation analysis to establish the relationship between all the variables.

## 4. Conclusion

In the present study, it was observed that the use of chicken dung (organic fertilizer) resulted in higher production of secondary metabolites and increased antioxidant activity compared to the use of NPK 15:15:15 (inorganic fertilizer). The production of total phenolics, flavonoid, ascorbic acid, saponin and antioxidant activity was highest under low nitrogen fertilization, especially at 90 kg N/ha. It was also observed that higher rates of fertilizer enhanced the production of nitrate. The nitrate content was found to be the lowest with chicken dung fertilization. The results indicated that the use of chicken dung was superior compared to the NPK fertilizer in producing high quality herbal plants (*L. pumila*) for pharmaceutical use. 
